# Quality of life in children with type 1 diabetes in Kuwait

**DOI:** 10.1186/1753-6561-6-S4-P7

**Published:** 2012-07-09

**Authors:** M Abdul-Rasoul, F AlOtaibi, M AlMahdi, H AlKandari

**Affiliations:** 1 Kuwait University, Kuwait

## Introduction

Recent research has shown that health-related quality of life (HRQOL) in children and adolescents with type 1 diabetes is markedly affected, resembling that of children with other chronic diseases, like malignancies. The objective of the study was to investigate the HRQOL in children and adolescents with diabetes in Kuwait.

## Methods

A total of 341 children and adolescents aged 5-18 years and 408 parents of children aged 2-18 years participated in the study. They were recruited from diabetes out-patient clinics in the 6 governorate hospitals. The pediatric quality of life inventory (PedsQL) questionnaire was used.

## Results

The mean (+/-SD) age of participants was 9+/-1.2 years, and the duration of diabetes was 4.9+/- 2 years. The Cronbach a coefficient of child and parent report generally approached 0.825, indicating their internal consistency and reliability. There was a statistically significant difference in the total scores among children and their parents in all 3 age groups (p < 0.001), however, to a lower degree in the adolescent group, where the main difference was in the "worry" section where parents reported worse QOL. The total scores showed good psychological adjustment of children and adolescents with diabetes, mean score (+/- SD_ was 85.7 (12.45), with slightly worse QOL in the 8-12 year old (71.2+/-13.1) p>0.05. Growing age, HbA1c, mode of insulin therapy, SES did not influence QOL of children with diabetes.

## Conclusions

Children and adolescents with type 1 diabetes and their parents in Kuwait showed good psychological adjustment and QOL. Parents appeared to be more worried than their adolescents about the effectiveness of the treatment and the long term complications.

